# Plant NLR immunity activation and execution: a biochemical perspective

**DOI:** 10.1098/rsob.230387

**Published:** 2024-01-24

**Authors:** Federica Locci, Jane E. Parker

**Affiliations:** ^1^ Department of Plant-Microbe Interactions, Max-Planck Institute for Plant Breeding Research, Carl-von-Linné-Weg 10, 50829 Cologne, Germany; ^2^ Cologne-Düsseldorf Cluster of Excellence on Plant Sciences (CEPLAS), 40225 Düsseldorf, Germany

**Keywords:** resistosome, Ca^2+^, ribosylated nucleotide, helper NLR, EDS1, TIR domain

## Abstract

Plants deploy cell-surface and intracellular receptors to detect pathogen attack and trigger innate immune responses. Inside host cells, families of nucleotide-binding/leucine-rich repeat (NLR) proteins serve as pathogen sensors or downstream mediators of immune defence outputs and cell death, which prevent disease. Established genetic underpinnings of NLR-mediated immunity revealed various strategies plants adopt to combat rapidly evolving microbial pathogens. The molecular mechanisms of NLR activation and signal transmission to components controlling immunity execution were less clear. Here, we review recent protein structural and biochemical insights to plant NLR sensor and signalling functions. When put together, the data show how different NLR families, whether sensors or signal transducers, converge on nucleotide-based second messengers and cellular calcium to confer immunity. Although pathogen-activated NLRs in plants engage plant-specific machineries to promote defence, comparisons with mammalian NLR immune receptor counterparts highlight some shared working principles for NLR immunity across kingdoms.

## Graphical abstract

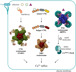

## 1. Introduction

Plant disease outbreaks remain the major threat to food production across continents, with losses caused by pathogens and pests reducing crop yields globally by approximately 30% each year [1]. New microbial strains which colonize and damage crops can now be detected more efficiently using high-throughput sampling, next-generation DNA sequencing technologies and epidemiology modelling [2]. Nevertheless, the spread of disease agents, exacerbated by global trade and a changing climate, presents a huge challenge for modern agriculture [1,3]. Insect borne *Xylella fastidiosa* bacteria threatening olive and grape production in southern Europe [4], the destructive *Puccinia graminis* f. sp. *tritici* wheat stem rust fungal Ug99 lineage spreading from Africa to Asia and the Middle East [5], and emergence of new strains of *Phytophthora* oomycete species infecting *Solanum* crops in South America and Europe [1,6] are examples of the many disease threats to food security.

Like animals, plants possess a genetically encoded ‘innate’ immune system to recognize microbes which have evolved invasion strategies to colonize host tissues and spread [7,8]. In both kingdoms, innate immunity pathways can be activated by both cell-surface and intracellular receptors detecting pathogen-derived ‘non-self’ molecules or pathogen-modified (damaged) host components [8–10]. Families of cell-surface receptors at the plasma membrane, referred to as pattern-recognition receptors (PRRs), intercept pathogen- or damage-associated molecular patterns (PAMPs or DAMPs). PRRs have ligand-binding ectodomains which transmit extracellular ‘disturbance’ to the cytoplasm. This then mobilizes defence cascades and nuclear transcription leading to pattern-triggered immunity (PTI) [9,11]. A further innate immunity layer is conferred by nucleotide-binding/leucine-rich repeat (NLR) receptors which detect pathogen interference inside cells [8,10]. NLR proteins belong to a large family of signal transduction ATPases with numerous domains (STANDs) [12]. Characteristically, NLRs possess C-terminal LRRs fused to a central nucleotide binding/oligomerization domain (NOD or NB) and various N-terminal portions which mediate defence signalling [10,13]. Whereas NLRs in mammals mainly detect intracellular PAMPs or DAMPs, the chief role of plant NLR receptors is as sensors of often variable virulence factors (called effectors) which are delivered inside host cells by infectious pathogen strains to promote infection [10]. Plant ‘sensor’ NLRs provide a crucial immunity barrier against host-adapted (virulent) pathogenic microbes by inducing a rapid defence process called effector-triggered immunity (ETI).

Tracing the origins and evolution of NLRs reveals that animals and plants have independently assembled structurally and functionally similar NLR multi-domain architectures from ancestral building blocks to serve the same purpose as immune- or cell death-triggering molecular switches [8,10,14–16]. Mammalian and plant NLRs are normally maintained in an inhibited (pre-activation) state through inter-domain interactions until specific ligand binding releases them from inhibition to trigger resistance and cell death pathways [10,13,17]. Hence, by their very nature, NLRs are dangerous molecules and defects that cause mis-regulation or mis-activation of NLR receptors can lead to autoimmunity with severe health and fitness consequences in both kingdoms [18–22].

In this review, we discuss progress made in understanding plant NLR functions and the roles of different NLR sub-types in immunity. Many genetically defined plant resistance (*R*) genes, found in natural populations and selected by plant breeders to confer disease resistance in crops, encode NLR proteins [23,24]. Over the last five or so years, our view of how NLRs operate as pathogen-activated molecular switches to counter disease has advanced considerably, building on solid genetic and molecular frameworks for host–pathogen interactions [24,25]. Here we examine some newly formulated biochemical principles for NLR-mediated pathogen surveillance and defence execution in host plants. The new information helps to explain how diverse immune receptors, recognizing pathogens with different attack strategies, converge on the same signalling machineries to promote an immune response.

## 2. Different evolutionary trajectories of plant and mammalian NLRs

Comparing mammalian and plant innate immune systems highlights some common working principles but also key differences between kingdoms. In mammals, the cellular innate immune response serves as an initial barrier to disease. PRRs and/or NLRs are engaged to induce pro-inflammatory cytokines and other immune-potentiating molecules which limit microbial infection [26]. Defence signals can be released through induced protein pores at the host plasma membrane, which enables communication with bystander cells for immune propagation [27,28]. Mammalian immune-related pore formation and signal release, and eventual regulated host cell death, help to prime the adaptive immune system with circulating antibodies that defend against specific pathogen strains [29].

Plants also use PRRs and NLRs but, in contrast to mammals, they rely entirely on their innate immune capability, and as sessile organisms, are under intense pressure to combat pathogenic microbes expressing suites of variable effectors [10,30]. These fundamental differences are reflected in numbers and diversity of immune receptor genes in these organisms [10]. In seed plants (angiosperms and gymnosperms), *NLR*s are the major characterized *R* gene determinants conferring ETI, although several valuable non-NLR based resistance mechanisms have been uncovered in crop species [31]. Sensor NLRs can be divided broadly into two sub-types which have different N-terminal signalling domains with distinctive signalling properties. Coiled coil-domain NLRs (CC-NLRs or CNLs) are present in dicotyledonous (such as *Arabidopsis* and other brassicas, potatoes, beans, cassava) and monocotyledonous (such as rice, wheat, barley, maize, banana) clades of the angiosperms [32]. By contrast, Toll/Interleukin-1 receptor/resistance protein (TIR)-domain NLRs (TIR-NLRs or TNLs) occur in the majority of dicot species but have been lost from monocots and a number of basal dicot lineages [15,21,33,34].

### 2.1. Expansion and contraction of plant immune receptor repertoires

Mammalian genomes generally show limited *NLR* gene expansion and variation, although some metazoans and vertebrates (such as sea urchin and zebra fish) have more extensive and diverse receptor panels [35,36]. Seed plant genomes encode hundreds to thousands of *NLR* genes [10]. Frequently, clusters of *NLR* variants reside in polymorphic loci that have arisen through tandem duplications and unequal cross-over events, as well as insertions and mutations [14,37,38]. Evidence suggests that NLR immune receptor repertoires of natural populations are considerably larger than in a single plant genotype, thereby maintaining useful receptor polymorphisms [39–42]. Evolutionary genomic studies have shown that plant immune receptor genes (*NLR*s and some *PRR* types) are among the most rapidly evolving of plant genes [41–44]. The LRR domains of different NLRs tend to display most variation, consistent with their role in variable pathogen effector recognition [30,45]. For instance, numerous allelic CNL receptor variants are encoded at barley *Mildew locus A* (*MLA*) resistance loci, each conferring immunity to a *Blumeria graminis* f. sp. *hordei* (powdery mildew) isolate delivering a matching AVR-Mla effector [46,47]. Some MLA variants recognize variable fungal effector epitopes presented on a common protein structural scaffold [48]. This might have facilitated pathogen effector escape from NLR recognition while maintaining virulence activity. Hence, plant hosts and adapted pathogens are in perpetual co-evolutionary conflict.

While certain NLRs, for example the barley MLA receptor variants, are activated through direct binding of a recognized effector molecule, NLR indirect effector sensing is also prevalent, especially in resistance to bacteria. This might be because many bacterial pathogenic effectors are enzymes which target host components [49]. Therefore, NLR-mediated indirect recognition is through sensing effector enzymatic activity rather than the effector itself [30]. Indeed, various modes of indirect recognition involve NLRs monitoring or ‘guarding’ the status of host defence components that are modified by particular pathogen effectors as part of their virulence strategy [30,50]. If the NLR-guarded host components (‘baits’ or evolved decoys of baits) are part of a defence hub targeted by different effectors, this would create an advantage for the host by broadening its NLR recognition ‘space’.

As discussed in the next sections, not all plant NLRs are variable pathogen effector sensors. Some are members of more conserved NLR families with roles in immunity and cell death signalling [21,32]. Conversely, not all PRR families bind conserved epitopes. For example, *Cladosporium fulvum* (*Cf*)-recognizing cell-surface receptor-like proteins (RLPs) in tomato intercept variable fungal effectors via their targeting of host papain-like cysteine proteases (PLCPs) in the plant apoplast [51,52]. Also, a phylogenetic study of cell-surface receptor-like kinase (RLK) family Pep-13 receptor unit (PERU) variants in South American wild potato populations revealed functional diversifying selection associated with PERU activation by its Pep-13 ligand from *Phytophthora* species [53]. Therefore, both intracellular and cell-surface immune receptor genes can evolve towards diversity or conservation [32].

### 2.2. Evidence for PTI and ETI concerted evolution in plants

Traditionally, plant cell-surface PRRs and intracellular NLRs were viewed as controlling two distinctive innate immunity layers (PTI and ETI, respectively). PRR recognition of patterns found in a broad class of microbes indeed limits colonization by non- or poorly adapted microbes [11]. Many functionally characterized pathogen-delivered effectors disable processes that promote PTI [25]. Direct or indirect NLR-effector recognition activates ETI which reinstates and strengthens PTI-related defence processes [25,54]. This often results in the death of host cells (micro-lesions) at attempted pathogen infection sites (called a hypersensitive response; HR). Recent genetic and functional studies in the model dicot plant *Arabidopsis thaliana* (hereafter *Arabidopsis*) show that there is extensive crosstalk between PTI and ETI receptor systems which mutually strengthens immunity outputs [55–57]. A functional convergence between PTI and ETI signalling machineries prior to nuclear transcriptional reprogramming would explain earlier findings that various *Arabidopsis* PTI and ETI responses produce qualitatively similar gene expression changes that differ more in speed and amplitude [25,54].

PTI–ETI coordination is likely to be broadly relevant, as a recent phylogenomic study reported a positive correlation between *PRR* and *NLR* gene numbers across land plant species [42]. Also, genomes of land plants that have acquired aquatic, parasitic or carnivorous lifestyles tend to carry fewer *NLR* and *PRR* genes [42,58,59]. Moreover, co-evolutionary pairing of functional (compatible) protein complexes between a sensor CNL HopZ-Activated resistance 1 (ZAR1) and co-functioning PTI-regulating HOPZ-ETI-DEFICIENT 1 (ZED1) cytoplasmic protein kinases appears to have arisen through altering pre-existing immunity modules [60,61]. Put together, these data suggest a functional basis for concerted gain and loss of cell-surface and intracellular receptor capacities as plants evolve and adapt to different niches.

## 3. Biochemical mechanisms of NLR activation

That the two major plant sensor NLR subtypes (TNLs and CNLs) share a multi-domain architecture was clear from the first cloned plant *CNL* and *TNL* receptor genes [10]. Molecular studies suggested a mechanism for CNL and TNL conformational activation mediated by their central adenosine diphosphate/adenosine triphosphate (ADP/ATP)-binding and exchange (nucleotide-binding adaptor shared by APAF-1, certain R gene products, and CED-4; NB-ARC) domains [62,63]. Further elegant studies of NLR domain functions revealed that amino acid variation in exposed LRR surface residues presented on a conserved leucine-rich scaffold underlie NLR-effector recognition specificity [47,63–65]. Researchers also identified key protein interfaces of isolated CC and TIR domains which mediate self-association and triggering of cell death when overexpressed in plants [66–68].

A much fuller appreciation of the activation principles of plant NLR receptors, and striking parallels with animal NLRs, emerged more recently from analyses of cryo-electron microscopy (cryo-EM) resolved NLR structures [24,69]. In both kingdoms, NLR activation through the C-terminal LRRs (or other repeat regions) drives ADP/ATP exchange in the plant central NB-ARC (or mammalian NB-NACHT) domain which leads to the formation of oligomeric signalling scaffolds, known as inflammasomes in mammals and resistosomes in plants. In these oligomeric complexes, the N-terminal domains are reoriented to be signalling-active [24,69]. In the following sections, we examine NLR N-terminal domain structures and modes of action in immunity.

### 3.1. A structural blueprint for sensor CNL activation and signalling

In 2015, the first reported cryo-EM structure of a pathogen-activated NLR was of a mouse inflammasome formed by the sensor NLR neuronal apoptosis inhibitory protein 2 (NAIP2) and a second signalling (or helper) NLR, NOD-, LRR- and caspase-associated recruitment domain (CARD)-containing protein 4 (NLRC4) following pathogen perception [70,71]. NAIP2 specific binding of components of the bacterial type III secretion system (such as a prgJ epitope) [72] leads to a conformational change which promotes oligomerization of NLRC4 protomers to form an ordered 10- or 11-mer wheel-like assembly with an unequal stoichiometry of 1:9 or 1:10 NAIP2:NLRC4 molecules [70,71]. In the NAIP2:NLRC4 hetero-oligomers, NLRC4 N-terminal caspase recruitment domains (CARDs) are organized to bind inflammatory caspase enzymes which then initiate pro-inflammatory signalling cascades leading to pathogen resistance and host pyroptotic cell death [69]. A different sensor NLR, NAIP5, promotes a similar NLRC4 inflammasome structure [73,74]. Also, NLR pyrin domain containing 3 (NLRP3) inflammasomes form a wheel-like homo-oligomer [75]. While in principle similar to the NAIP2-NLRC4 and NAIP5-NLRC4 inflammasomes, NLRP3 senses cellular and membrane homeostasis and requires centrosomal NIMA-related kinase 7 (NEK7) as well as an apoptosis-associated speck-like protein containing a CARD (ASC) adaptor to recruit caspases and induce pyroptosis [8,75].

**Figure 1.**
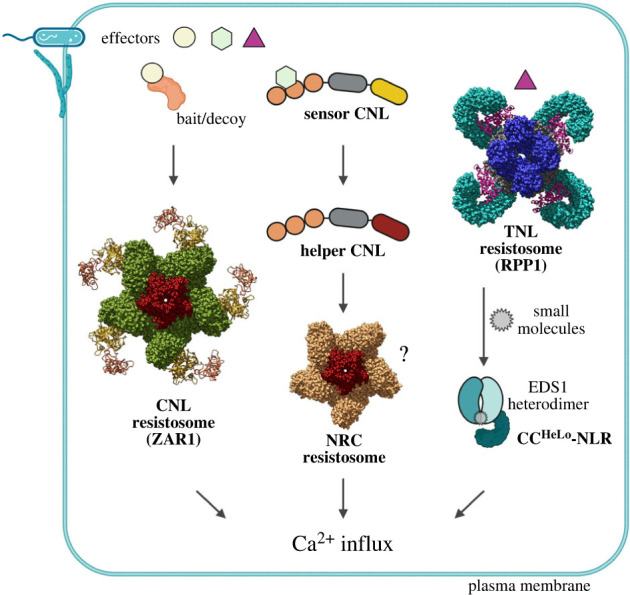
Different plant NLR resistosomes converge on Ca^2+^ influx in immunity. Left and centre: sensor CNLs are conformationally activated by direct or indirect effector recognition, which leads to formation of a pentameric CNL resistosome. The CNL resistosome has a funnel-like structure (scarlet) which creates an autonomous Ca^2+^-permeable channel at the plasma membrane to promote immunity and cell death by increasing Ca^2+^ influx to the cytoplasm. CNLs of the NRC family serve as ‘helper’ or signalling NLRs following effector perception by sensor CNLs, which are not part of an NRC resistosome-like complex. Induced NRC oligomers might also be plasma membrane-bound Ca^2+^-permeable channels potentiating immune responses. Right: direct effector detection by a sensor TNL (eg. RPP1) leads to assembly of a tetrameric TNL resistosome with TIR-domain (dark blue) encoded NADase activity. The induced TNL NADase enzyme generates nucleotide-based small molecules, some of which are bound by EDS1 heterodimers to conformationally induce their association with co-functioning CC^HeLo^-domain helper NLRs. EDS1 dimer-activated CC^HeLo^-NLRs likely also mobilize immunity by forming resistosome-like membrane channels that promote Ca^2+^ influx into cells. (Displayed CNL pentameric resistosome is based on ZAR1 structure PDB: 6J5T. TNL resistosome is based on RPP1 structure PDB: 7CRC). Three-dimensional models were generated with UCSF Chimera software (www.rbvi.ucsf.edu/chimera). Figure was generated with Biorender.com.

In 2019, cryo-EM approaches enabled the structural characterization of pre-activated monomeric and pathogen-activated forms of the *Arabidopsis* CNL receptor ZAR1 [76,77]. Determining the structural organization of both ZAR1 states (an auto-inhibited CNL monomer and active oligomer) revealed how, in this case, indirect bacterial effector recognition generates a signalling-active ZAR1 homo-pentamer [76,77] (figure 1, left). These studies were hugely significant in part because they revealed a shared general working principle for NLR activation between plants and animals. Assembly of the ZAR1 resistosome promotes its association with the plasma membrane and exposes the five NLR CC domains with realigned N-terminal α-helices to form a non-selective calcium (Ca^2+^)-permeable ion channel [77,78] (figure 1, left). The cryo-EM structure and functional characterization of a second plant sensor CNL resistosome, that of the wheat stem rust resistance protein Sr35, revealed a similar homo-pentameric architecture to ZAR1 and Ca^2+^ ion channel activity [79,80]. In contrast to ZAR1, the LRR domain of Sr35 directly binds its cognate fungal effector AvrSr35. These two CNL resistosome assemblies therefore suggest a pentameric blueprint for activated sensor CNLs in dicot and monocot species, irrespective of whether the CNL is directly (Sr35) or indirectly (ZAR1) activated by a pathogen effector. Comparing NLR amino acid coordinates required for ZAR1 and Sr35 oligomerization suggests a common CNL mode of action in which an LRR conformational shift caused by the pathogen creates a stearic clash between the LRRs and ADP-bound NB-ARC domain, thereby releasing the CNL protein from inhibition [79,80]. In this model, ADP is readily exchanged by ATP which, as it becomes hydrolysed [63], drives further conformational changes that stabilize the resistosome pentamer. Additionally, the ZAR1 and Sr35 studies provided evidence that CNL resistosome ion channel activity at the plasma membrane is a necessary step for promoting cell death and pathogen resistance in ETI (figure 1).

Presumably, CNL activation in host cells receiving a recognized pathogen effector and CNL resistosome-mediated Ca^2+^ influx to the cytoplasm provide a stimulus for Ca^2+^-dependent signalling cascades, such as those mediated by Ca^2+^-dependent protein kinases and transcription factors known to orchestrate ETI resistance and localized cell death [81–83]. Because the *Arabidopsis* ZAR1 and wheat Sr35 oligomers induced Ca^2+^ influx in cultured *Xenopus* oocytes [78,79], it is thought that CNL resistosomes represent an entirely new type of autonomous ion channel in plants. Remarkably, a small protein, WeiTsing (WTS encoded by the *Arabidopsis* *Resistance to Plasmodiophora brassicae 1 RPB1* gene [84]) expressed in *Arabidopsis* roots and unrelated to CNLs or other known plant ion channels, was reported to confer broad spectrum resistance to the *Plasmodiophora brassicae* pathogen causing club-root disease by forming a pentameric Ca^2+^-permeable ion channel at endoplasmic reticulum membranes [85]. How Ca^2+^ influx into the cytoplasm by CNL resistosomes or a WTS membrane channel are coordinated with canonical ion channel activities known to contribute to immunity remains unclear [81–83].

### 3.2. Functional CNL sensor and helper networks

Whereas *Arabidopsis* ZAR1 and wheat Sr35 appear to behave as singleton sensor NLRs working, as it were, alone as Ca^2+^-permeable ion channels to induce ETI defence and cell death, other plant CNLs have been characterized genetically and functionally which cooperate as interacting sensor–helper NLR pairs to confer disease resistance [32]. Well-studied examples of co-functioning CNL pairs are rice RGA5 with RGA4 and Pik-1 with Pik-2 in which the sensor NLR (RGA5 or Pik-1) in some way transmits effector activation through a conformational change to the helper NLR (RGA4 or Pik-2) within a stable hetero-complex to mobilize resistance [86–88]. Such sensor–helper CNL pairs might form a two-tier hetero-pentameric complex with ion channel activity at a cell membrane, although other CNL induced configurations with different immunity outputs are possible.

A further mode of CNL sensor–helper cooperation was discovered from analysis of functional networks between various solanaceous sensor CNLs and a related family of NLR-required for cell death (NRC) signalling or helper CNLs [89] (figure 1, centre). In *Nicotiana benthamiana,* different sensor CNLs utilize four NRC1–NRC4 helper CNL paralogues in a partially overlapping manner to signal pathogen resistance and host cell death [89–91]. NRC3 and NRC4 proteins have predicted CC-domain N-terminal *α*1-helices which, for NRC4, were functionally interchangeable with ZAR1 in cell death assays [91]. Therefore, NRCs might also behave as membrane-bound ion channels. Two tested effector-activated sensor CNLs (wild potato Rpi-amr3 recognizing effectors produced by *Phytophthora infestans* or Rx recognizing the coat protein of potato virus X) genetically required *NRC2* and *NRC4* for pathogen resistance and host cell death [17,92]. However, the activated sensor CNLs did not stably associate with their co-functioning NRC proteins in plant transient assays (figure 1, centre). Instead, NRC2 and NRC4 each accumulated as a high molecular weight complex *in vivo* [17,92]. Collectively, the data suggest that NRC-recruiting sensor CNLs transmit a change in their status and/or conformation in a transient manner which facilitates the helper CNL to then assemble into a homomeric ZAR1-like resistosome with possible ion channel activity at the plasma membrane [17,92] (figure 1, centre). An ‘activation and release’ model was proposed for certain sensor CNL receptors that signal via NRC helpers [32]. It might be that non-inclusion of a sensor NLR in an NRC-type resistosome is energetically favourable for defence signal propagation, especially when the initial sensor NLR or cell-surface receptor stimulus is weak [24]. It seems that Ca^2+^ influx might also be an output for NRCs, with NRCs serving as Ca^2+^ channels or pores induced by endogenous host signals. A network of helper CNLs operating together with immune sensors that do not take part in pore or channel formation might provide flexibility for the sensor to evolve new recognition surfaces in response to pathogen effector pressure [32,93].

While the molecular relationships between sensor and helper CNLs appear to vary, current models depict activated sensor and helper CNLs converging on resistosome-like complexes with Ca^2+^-permeable ion channel activities to promote ETI resistance and cell death (figure 1, left and centre). This is a very different NLR output from that described for mammalian inflammasomes (§3.1), even though a guiding principle for animals and plants is that NLR conformational activation promotes the assembly of oligomeric protein scaffolds for downstream signalling [24,69]. It remains unclear whether plant CNL and structures functions are restricted to assembly of Ca^2+^-permeable pores or channels at the plasma membrane. Since nuclear localization is required for immunity activities of some CNLs [94], CNLs might directly regulate transcriptional programming in the nucleus by interacting with transcription factors [95]. Conceivably, some CNLs might form Ca^2+^ channels at the nuclear membrane or endoplasmic reticulum [96,97]. This could generate signals for speedy transcriptional changes and eventual cell death at infection sites.

### 3.3. Pathogen-activated TIR-domain NLR resistosomes are NADase enzymes

A structurally compact TIR domain located at the N-terminus of the plant TNL receptor sub-class has immune-related functions in all cellular kingdoms of life [98,99] (see also §§4.1 and 4.2). In mammalian immunity, TIR-containing protein modules work principally as signalling adaptors which, through TIR–TIR self-association, integrate cell-surface PRRs with intracellular defence cascades to mobilize transcription and cell death [98,100]. Characterization of TIR-containing human protein sterile alpha and Toll/interleukin-1 receptor motif-containing 1 (hSARM1) revealed a new TIR biochemical function as an NAD^+^ hydrolysing enzyme regulating neurodegeneration [99,101–103]. A metabolically induced conformational change in hSARM1 leads to TIR–TIR associations as a two-stranded assembly [104], creating an active NADase enzyme which produces at least one bioactive cyclic ADP-ribose (cADPR) intermediate and depletes cellular NAD^+^ to promote axonal cell death [103,105].

The TIR catalytic mode of action in hSARM1 prompted a redefining of certain TIR-domain proteins as metabolic regulatory enzymes [106]. Investigations of plant and bacterial TIR-domain proteins showed that several indeed have NADase activity leading to cell death in *N. benthamiana* transient expression assays, and requiring a conserved TIR glutamic acid residue that is shared with hSARM1 [68,106–108]. The discovery that some plant TIR domains are NADases with a capacity to generate ribosylated cyclic nucleotide products *in vitro* and *in vivo* raised the notion that a similar enzymatic action might underlie TNL receptor signalling. The cryo-EM structures of two TNL resistosomes—*Arabidopsis* Recognition of *Peronospora parasitica* 1 (RPP1) and *Nicotiana benthamiana* Recognition of XopQ 1 (Roq1)—both activated directly by cognate pathogen effector binding to the LRR domains [65,109], revealed the biochemical mechanism of TNL activation leading to the assembly a TIR-domain NADase enzyme [110,111] (figure 1, right). The pathogen-activated RPP1 and Roq1 resistosomes have a similar homo-tetrameric TNL architecture in which four TIR domains are orientated as two asymmetrically aligned pairs to create two composite NADase catalytic sites required for TNL signalling [111]. As pathogen-activated NADase enzymes, TNL resistosomes thus have different immediate signalling properties to CNL resistosomes, despite both TNL- and CNL-triggered immunity converging on similar transcriptional defence programmes [54,112].

## 4. TNL receptor signalling and immunity execution

**Figure 2.**
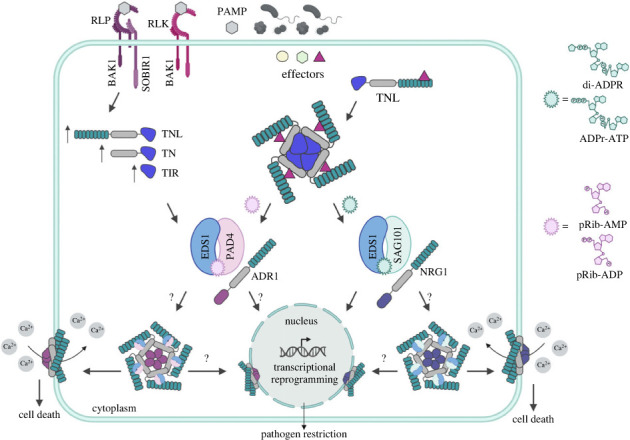
TIR-domain enzymatic activities at the heart of PTI and ETI responses. Two sets of TIR-domain catalysed ribosylated nucleotides conformationally activate two related but functionally distinct EDS1 heterodimer complexes. Specific nucleotide binding to EDS1-PAD4 or EDS1-SAG101 complexes promotes their association, respectively, with ADR1- or NRG1-family CC^HeLo^-NLRs. The EDS1 dimer-activated ADR1 or NRG1 probably form CNL resistosome-like pentamers with Ca^2+^-permeable channel activities at the plasma membrane. Evidence suggests additional nuclear EDS1 dimer–CC^HeLo^-NLR pools contribute to the immune response either as a Ca^2+^-permeable ion channel/pore at the nuclear membrane (shown) or as a functionally different nuclear complex promoting transcriptional reprogramming. In *Arabidopsis*, mutual defence potentiation between cell-surface PRR-triggered immunity (PTI) and intracellular effector-triggered immunity (ETI) machineries might be facilitated by transcriptionally or otherwise mobilized TNL- and TIR-only enzymes generating phosphoribosylated nucleotide intermediates pRib-ADP/AMP and ADPr-ATP/di-ADPR which, respectively, activate the EDS1-PAD4-ADR1 and EDS1-SAG101-NRG1 immunity branches. Question marks (?) indicate putative routes to transcriptional reprogramming. Figure was generated with Biorender.com.

The biochemical insights into plant CNL and TNL resistosome activation mechanisms described in §3 provided a fresh impetus to dissect molecular processes linking pathogen effector recognition to induced host defence and cell death in ETI. Clues to CNL resistosome modes of action as oligomeric Ca^2+^ channels at plant membranes (figure 1, left and centre; §3.1) have been discussed. Here we consider evidence for pathogen-activated oligomeric TNL receptors with NADase activity also converging on CNL-related Ca^2+^ outputs in ETI (figure 1, right; figure 2).

### 4.1. Pathogen-activated TNL receptors utilize two CC^HeLo^-NLR branches to promote immunity

Besides the TIR-domain NLR receptors (TNLs) conferring ETI in dicot species, plants also express smaller TIR-NB and TIR-only proteins with immune-related activities [34,113–116]. In plant transient expression assays, individual TIR domains taken from various TNLs or a natural truncated TIR-domain Resistance protein (*Arabidopsis* RBA1) induced cell death [34,107,108,113,117]. Tested TIRs required TIR–TIR self-association and an intact NADase catalytic site to generate NAD^+^ hydrolysis products nicotinamide (NAM) and two cyclic ADPR variants *in vitro* and *in vivo* [107,108]. Therefore, TNL- and TIR-generated NAD^+^ catalytic products emerged as possible signalling intermediates for pathogen resistance and cell death execution in plants.

Genetic dissection of defence pathways in *Arabidopsis* and *N. benthamiana* identified two plant-specific protein families which transduce TNL and TIR NADase-generated signals leading to pathogen resistance and host cell death. The first family comprises three lipase-like immune regulators consisting of enhanced disease susceptibility 1 (EDS1), senescence associated gene 101 (SAG101) and phytoalexin deficient 4 (PAD4), which regulate TNL ETI responses to recognized pathogen strains and basal immunity responses to colonizing pathogens [21] (figure 2). Basal immunity is thought to be the combined outcome of partially disabled PTI (after effector interference) and weak ETI, since it limits colonization by virulent pathogens without inducing host cell death [118]. *Arabidopsis* EDS1 forms exclusive heterodimers with SAG101 or PAD4 through non-catalytic binding surfaces in the partner N-terminal lipase-like domains [119]. This draws together EDS1-SAG101 or EDS1-PAD4 C-terminal α-helical bundle domains to create two heterodimer surfaces mediating, respectively, TNL ETI and basal immunity signalling [119–121].

The second family consists of two CNL-like subgroups of signalling (or helper) NLRs: N requirement gene 1 (NRG1) and activated disease resistance 1 (ADR1), with 4-helical bundle HET-S/LOP-B (HeLo) (CC^HeLo^ or CC^RPW8^) N-terminal domains [122,123] structurally resembling the CC domains of CNLs [124]. Notably, the CC^HeLo^ topology is present in a range of (non-NLR) immunity and cell-death regulators in fungi, plants and mammals, consistent with this domain being recruited for immune and/or cell death signalling across kingdoms [28,123]. As far as phylogenomic data tell, both the EDS1- and CC^HeLo^ -NLR families evolved at an early stage of seed plant speciation and therefore post-date the origins of TNL and CNL immune receptor genes [21,37,125–127]. Hence, EDS1-family proteins and CC^HeLo^-NLRs represent plant-specific machineries engaged for immunity signalling.

Further genetic and protein structure-based studies in *Arabidopsis* and *N. benthamiana* revealed that EDS1 and SAG101 cooperate with NRG1s (of which there are two functional homologues in *Arabidopsis*) in a single immunity signalling branch (or node) which promotes TNL ETI-associated transcriptional defences and host cell death [123,128–130] (figure 2). A second TNL-triggered branch formed by EDS1 and PAD4 in cooperation with ADR1s (three functional isoforms in *Arabidopsis*) mobilizes transcriptional defences which help to restrict pathogen growth in ETI and basal immunity [129,131,132] (figure 2). The components of each immunity branch were found to be non-interchangeable, genetically and in plant reconstitution assays [33,128,129,133], pointing to a tight functional relationship between individual EDS1 heterodimers and their specific CC^HeLo^-NLR sub-types. Thus, EDS1-SAG101-NRG1 and EDS1-PAD4-ADR1 constitute distinct immune signalling branches [21].

A co-occurrence pattern of *TNL*, *SAG101* and *NRG1* orthologues in seed plant lineages supports a dedicated role of the EDS1-SAG101-NRG1 node in TNL-triggered immunity, restricted to dicot plants [33,59]. By contrast, EDS1-PAD4-ADR1 node genes are present in all examined seed plant genomes, including monocots and several basal dicot clades which lack *TNL* genes but retain truncated *TIR-only* and *TIR-NB* genes [33,34,59]. This wider *PAD4* and *ADR1* phylogenetic distribution fits with broader roles established in *Arabidopsis* for the EDS1-PAD4 dimer and ADR1s in ETI transcriptional defence potentiation conferred by TNLs and CNLs, and PTI triggered by certain cell-surface PRRs [43,118,120,128,132,134]. Notably, PTI in *Arabidopsis* also leads to the rapid upregulation of several *TNL*, *TIR-NB* and *TIR-only* genes [134] (figure 2). ETI–PTI cross-potentiation [55,56] therefore probably lies, at least in part, with convergence of TIR-generated nucleotide signals on the EDS1-PAD4-ADR1 immunity branch [118].

### 4.2. EDS1 dimers are receptors for TIR-generated ribosylated nucleotides

EDS1-SAG101 and EDS1-PAD4 dimers possess similar positively charged grooves formed by the partner C-terminal domain α-helices [119]. In *Arabidopsis*, positionally equivalent amino acid residues in each dimer groove determined both their induced associations with co-functioning CC^HeLo^-NLR sub-types (NRG1s versus ADR1s) and distinctive EDS1-SAG101 and EDS1-PAD4 immunity contributions (TNL ETI-related cell death versus basal immunity) [33,121,128,135] (figure 2). These findings cemented the idea that EDS1-SAG101 and EDS1-PAD4 complexes bind similar TNL/TIR enzymatic nucleotide products to promote CC^HeLo^-NLR association and, thereby, immunity execution.

The above model was realized through a series of reconstitution experiments performed with insect cells. Co-expression of *Arabidopsis* NRG1 or ADR1 proteins together with the NADase-active *Arabidopsis* TNL (RPP1) resistosome and *Arabidopsis* EDS1-SAG101 or EDS1-PAD4 complexes in insect cell cultures revealed that the two EDS1 dimer types indeed bind TIR NADase products [136,137]. The TIR-generated nucleotides stabilize EDS1 dimer interactions with their co-functioning CC^HeLo^-NLRs, thereby recapitulating interaction specificities observed in plants (figure 2). Through small molecule biochemical analyses and protein structural determinations, it was established that the TIR domains of a tetrameric TNL resistosome [111], and similarly orientated TIR-only proteins, undergo an ADP-ribosyl transferase reaction using NAD^+^ or NAD^+^ with ATP as substrates to generate, respectively, di-ADP-ribosylated (di-ADPR) and ADP-ribosylated ATP (ADPr-ATP) as non-cyclic nucleotide signals [137]. ADPr-ATP or di-ADPR binding by EDS1-SAG101 dimers at sites along the C-terminal groove leads to a SAG101 conformational change which promotes NRG1 association [137] (figure 2).

EDS1-PAD4 binding of two less bulky TIR NADase products, 2′-(5″-phosphoribosyl)-5′-adenosine mono-/di-phosphate (pRib-AMP and pRib-ADP), in the dimer groove induces a similar conformational change in PAD4 leading to its stable association with ADR1 [136] (figure 2). *In vivo* assays of mutated EDS1 dimer variants confirmed that intact nucleotide binding sites in the two *Arabidopsis* EDS1 heterodimers are necessary for their respective immunity outputs [33,120,128,135]. Importantly, key amino acid residues coordinating nucleotide binding and *Arabidopsis* EDS1 dimer–CC^HeLo^-NLR associations *in vitro* were found to be conserved in EDS1-family orthologues across seed plant species [136,137]. This suggests that specific interactions between EDS1 dimers and TIR catalytic products is a broadly relevant mechanism for activating CC^HeLo^-NLR mediated immune responses. The model is supported by *in vitro* assays which showed that a monocot TIR-only protein from the grass species *Brachypodium distachyon* (*Bd*TIR) also promotes EDS1-SAG101-NRG1 or EDS1-PAD4-ADR1 specific associations in a TIR NADase-dependent manner [136,137]. Hence, two sets of TIR-generated ribosylated nucleotides were proposed to represent a new class of immune second messenger linking enzymatic TNLs and TIRs to defence and cell death in plants [99,138].

### 4.3. CC^HeLo^-NLRs promote immunity downstream of TNL-activated EDS1 dimers

A new picture emerges in which EDS1 dimers, as receptors for two sets of TIR- and TNL-produced ribosylated nucleotide, act as host activators of helper NLRs, in this case CNL-like CC^HeLo^-NLR proteins (figure 2). The molecular and functional relationship between TIR small molecule-modified EDS1 dimers and CC^HeLo^-NLRs might, in principle, resemble bacterial effector indirect activation of the sensor CNL ZAR1 through modification of host proteins, which leads to ZAR1 pentamerization and Ca^2+^ ion channel activity [77,78] (see also §3). If this is the case, the EDS1–CC^HeLo^-NLR nodes provide a means to connect diverse sensor TNLs in ETI and induced TNLs and TIRs in PTI to potentially similar Ca^2+^-dependent outputs as the ZAR1 and Sr35 CNL receptors [24,138]. Although ADR1s and NRG1s are phylogenetically distinct from CNLs, they do oligomerize and associate with the plasma membrane in their activated forms [124,131,133,139] (figure 2). It is interesting that combined ETI and PTI stimuli were needed to detect NRG1 resistosome-sized oligomers in *Arabidopsis*, consistent with PTI boosting the production of TIR- and TNL-generated nucleotides to potentiate ETI [139].

Accumulating data therefore suggest a quite simple model in which TIR-activated EDS1-family receptors promote the assembly of CC^HeLo^-domain pentameric resistosomes with Ca^2+^-permeable ion channel activities at the plasma membrane or endomembranes (figure 2). Nevertheless, an EDS1 dimer-activated CC^HeLo^-NLR structure is still lacking. Also, this model does not explain reported requirements for EDS1 and SAG101 nuclear accumulation in TNL immunity and a detected EDS1-SAG101-NRG1 nuclear pool in ETI-activated cells [139–141]. Conceivably, EDS1-mobilized helper NLR nuclear complexes could release Ca^2+^ directly into nuclei by forming channels at the nuclear membrane. Alternatively, these components might have different or additional, yet unknown, nuclear activities in regulating transcriptional defence (figure 2). Whatever the underlying mechanism, a nuclear EDS1-SAG101-NRG1 pool could enable fast transcriptional reprogramming for pathogen containment [129,130] in host cells that directly receive TNL-recognized pathogen effectors to produce EDS1/CC^HeLo^-NLR activating nucleotide signals. EDS1-PAD4 and ADR1 pools could then potentially mop up TNL- and TIR-generated ribosylated nucleotides in surrounding plant cells and tissues to reinforce ETI and spread basal defences [126]. The temporal and spatial dynamics of ribosylated nucleotide synthesis, persistence and bioactivity are not understood, but likely depend on the availabilities of active TNL and TIR enzymatic modules as well as their essential downstream components for signal relay and defence execution.

### 4.4. Bioactivities of TIR-domain NADase products

The TIR domain is an intriguingly versatile enzymatic module contributing to immunity signalling in animals, plants and bacteria [98,99,138]. The discovery and structural characterization of hSARM1 revealed that it functions as a ligand-regulated TIR-encoded NAD^+^ hydrolysing enzyme [102,104–106] (§3.3). This produces a NAD^+^-derived product, cADPR, which together with NAD^+^ depletion, promotes intra-axonal Ca^2+^ fluxes from intracellular and extracellular calcium stores and contributes to axonal degeneration [103].

In bacteria, an anti-phage resistance mechanism called ‘Thoeris’ (Ths) has been elucidated which requires a sensor TIR-containing protein, ThsB, and a non-TIR sirtuin2-type (SIR2) NADase, ThsA, both with the capacity to cleave NAD^+^ [142]. By hydrolysing NAD^+^, ThsB produces a cyclic ADPR isomer, 3′-cADPR, which binds with high potency to a pocket in ThsA, thereby promoting ThsA NADase activity and host cell death through the depletion of cellular NAD^+^ [117,143,144]. This host cellular response stops the spread of phage infection. A different bacterial NAD^+^ derived cADPR isomer, 2′-cADPR, was identified as an *in vitro* and *in vivo* product of a plant-infecting *Pseudomonas syringae* TIR NADase effector, HopBY, and delivery of HopBY induced disease-like symptoms in *Arabidopsis* [145]. Both 2′-cADPR and 3′-cADPR were reported products of resistance-dampening *P. syringae* TIR-domain NADase effectors [117,145,146]. Therefore, 2′-cADPR and 3′-cADPR might have immune suppressive roles in plants. Supporting this model, *P. syringae* effector HopAM1, which generates 3′-cADPR *in vivo* [146], failed to promote an EDS1-PAD4 interaction with ADR1 *in vitro* [136]. Similarly, a TIR NADase *Ab*TIR from *Acinetobacter baumannii* bacteria which produces 2′-cADPR did not elicit *EDS1*-dependent cell death in *N. benthamiana* [68]. These data point to inhibitory activities of TIR-catalysed 2′-cADPR and 3′-cADPR molecules in immune responses of plants and possibly other host organisms.

Plant TIR domains can display other catalytic properties. For example, *Arabidopsis* TIR-only protein Response to HopBA1 (RBA1) [115,117] and the TIR domain of *Linum usitatissimum* (flax) TNL receptor L7 [64] were found to have a combined nuclease and cyclic nucleotide synthase activity when presented with a double-stranded RNA or DNA substrate *in vitro* [147]. Formed TIR–nucleic acid interfaces produced a TIR filament-like assembly from which the TIR domains generated 2′,3′-cyclic AMP/GMP [147]. These cyclic molecules represent a different set of nucleotide-based signalling intermediates with roles in stress potentiation. The filament-forming TIR domains with nuclease/cyclic synthetase activity are oriented differently from the asymmetric TIR pairs of TNL resistosomes with NADase/ADP-ribosyl transferase activity [110,147]. Thus, TIR domains appear to be versatile enzymes with a capacity to produce a range of immune- and stress-stimulating nucleotide signals.

## 5. NLRs working between local and systemic immunity

In contrast to mammals, plants can develop new organs when damaged. Therefore, localized infections are not a major threat unless they spread. As discussed, mammalian and plant NLR activation is often associated with regulated cell death [28]. Cell death responses at local infection sites help to shut off nutrient supplies to biotrophic or hemi-biotrophic pathogens and instruct bystander cells to mobilize anti-microbial defences which restrict disease progression [28,126]. In animals, local ‘danger’ signals are perceived and amplified by surrounding immune cells via pro-inflammatory molecules, such as cytokines and chemokines, which together with Ca^2+^ and H_2_O_2_ are released through induced pores at the plasma membrane to prime other immune cells for defence [26,148]. In plants, the local-to-distal transmission of immune signals is a different challenge, as cells have walls and are fixed in tissues. Communication between plant cells occurs in the apoplast (consisting of cell walls, intercellular spaces and the vasculature) and the symplast (a cytoplasmic continuum between cells connected by plasmodesmata) [149]. Both routes are used to transmit immune and damage signals from locally infected to systemic tissues [149,150].

**Figure 3.**
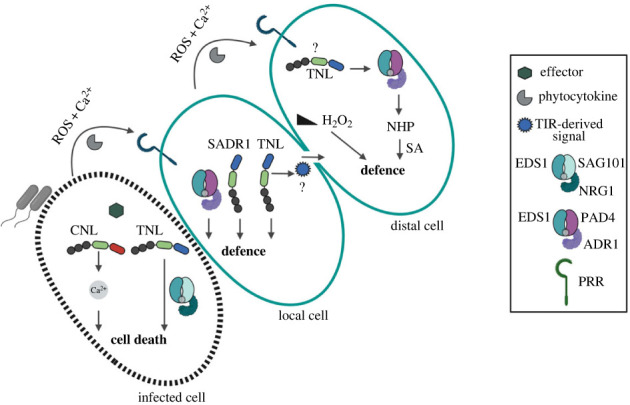
A broader role for TNLs in local to systemic immunity transmission. The scheme depicts a model for the spatial regulation of NLR-triggered immunity promoted by TNL-activated EDS1-family dimers with CC^HeLo^-domain helper NLRs. In cells directly responding to pathogen attack through effector recognition (infected cell), sensor CNLs (autonomously or with CNL helpers) and sensor TNLs (via the EDS1-SAG101-NRG1 node) promote an ETI response leading to death of the responding cell. This reaction produces immunogenic signals and resistance-potentiating ROS and Ca^2+^ waves. Bystander cells (local cell) in the vicinity of the ETI-triggered cell perceive mobile defence signals such as endogenously generated peptides (phytocytokines) recognized by cell-surface receptors. These reactions create further defence propagation via the EDS1-PAD4-ADR1 node, the TNL SADR1 and likely other TNL and TIR-domain protein activities. EDS1-PAD4-ADR1 also promotes immunity in distal tissues (distal cell) through NHP and salicylic acid (SA) synthesis, together with actions of a ROS gradient (H_2_O_2_). How EDS1-PAD4-ADR1 would be activated in distal tissues is not known. Further TNLs and TIR-domain proteins might help to transduce and propagate immunogenic signals in systemic tissues. It is also possible that immunogenic molecules generated by locally triggered cells travel via the symplast (plasmodesmata cell-to-cell connectors) to activate defence in more distal cells. Figure was generated with Biorender.com.

The best characterized mobile and/or distally produced immune signals in plants are an induced stress metabolite, *N*-hydroxypipecolic acid (NHP), and the biotic stress phytohormone salicylic acid (SA) [117,150–152] (figure 3). Additionally, host apoplastic proteins and peptides called phytocytokines are important signals for transmission of immune and damage responses between cells and tissues [153,154]. In dicots and monocots, phytocytokines act as danger signals coordinating phytohormone and other stress or developmental responses, but not cell death [153,155]. A well-studied phytocytokine is *Arabidopsis* pep1 which is generated upon leaf wounding or pathogen infection and plays a key role in defence transmission between cells, together with defence-propagating Ca^2+^ and reactive oxygen species (ROS) waves [156–158] (figure 3). A recent preprint reports a role for H_2_O_2_ gradients generated by locally infected cells in transcriptional mobilization of systemic immunity through post-translational modifications of transcription factors controlling SA signalling and systemic defence [159] (figure 3). Thus, cell-to-cell resistance propagation in plant tissues appears to involve an intricate circuitry of mobile signals and defence amplifying loops, which are only partially understood.

### 5.1. TNLs contribute to local and distal defence propagation

Contributions of TNL and TIR enzymatic activities to plant local-to-distal defence relay seem likely (§4). In ETI-responding tissues, the TNL-triggered EDS1-PAD4-ADR1 signalling branch transcriptionally potentiates SA-dependent and SA-independent basal defences [120], and promotes systemic immunity [126] (figure 3). Also, *EDS1* and *PAD4* are genetically required for NHP generation in distal tissues [152]. Current evidence positions EDS1-PAD4-ADR1 in a zone bordering ETI-stimulated dying cells where it transcriptionally regulates SA, NHP and other defence pathways [126,160] (figure 3). The availability of TNL- and TIR-generated pRib-AMP/ADP nucleotides, as well as EDS1-PAD4-ADR1 node components that these molecules activate inside cells, will likely determine the effectiveness and spread of defences around ETI foci [161].

Although TNLs have been characterized principally as pathogen effector-sensing devices with direct ETI roles in cells that are destined to die, a suite of *TNL* and *TIR*-domain genes are upregulated in cell-surface receptor mediated PTI [34,134], consistent with TNL and TIR enzymatic activities (§4) also stimulating immune responses in non-dying cells bordering infection sites. A recent study identified a TNL protein Suppressor of ADR1-L2 1 (SADR1) in *Arabidopsis* which promotes defence gene expression and pathogen containment in cells around infection sites, but is dispensable for tested ETI responses [161] (figure 3). SADR1 appears to function as a canonical NADase enzyme in this action but signals in a partially *EDS1*-independent manner [161] (figure 3). These data highlight a TNL NADase contribution to defence potentiation in a localized zone surrounding bacterial infections. How then are SADR1 and potentially other TNLs in bystander cells activated? One important factor might be the provision of cytoplasmic Ca^2+^ through channels, since Ca^2+^ ions stimulate TNL NADase activity *in vitro* [111]. Another might be TNL post-translational modifications. Phosphorylation was reported to control the activation of an *Arabidopsis* TNL receptor pair, RRS1-RPS4 [162], and therefore might regulate recruitment of TNLs for cell-to-cell defence signalling. Strikingly, *TNL* genes were found to be strongly induced in cells of the plant vasculature following fungal infection [163], suggesting involvement of TNLs in apoplastic signal propagation. It is also possible that TNL- and TIR-generated nucleotides produced in pathogen-activated cells are transported to other cells via the symplast, as part of an EDS1-PAD4-ADR1 defence propagating loop (figure 3).

## 6. Conclusion and outlook

We have examined some new working principles in plant NLR immunity, derived mostly from biochemical and structural insights into protein functions. Conceptual parallels with mammalian NLRs can be seen at the level of building oligomeric protein complexes to trigger immune responses, and the importance of cell-to-cell defence propagation. Three discoveries in plants seem pivotal to moving the field forward. The first is that pathogen-activated sensor CNLs and TNLs form two types of resistosome with different signalling properties. The second is that CNL resistosomes are plasma membrane Ca^2+^ permeable channels. Ca^2+^ channel activities of sensor and helper CNL-type NLRs might therefore define immune signal relay within and between cells. The third is that a set of TNL- and TIR- protein-generated ribosylated nucleotides connect cell-surface and intracellular receptor systems to immunity execution by activating two EDS1-family/CC^HeLo^-NLR signalling branches. Collectively, these findings provide a much clearer picture of the plant defence network and fresh leads for engineering disease resistant crops.

Nevertheless, there are important knowledge gaps to fill in future studies. For example, it remains unclear how the described NLR-generated Ca^2+^-permeable channels, whether pathogen- or host-activated, are coordinated temporally and spatially with other immune-related Ca^2+^ ion channel families. This seems fundamental to understand the dynamics of Ca^2+^ signalling within and between host cells and whether different channels employ the same or different decoders to transcriptionally reprogramme cells for defence and cell death. Also, the issue that some sensor and helper NLRs localize to the nucleus remains unresolved. Is a nuclear location compatible with presumed CNL and CC^HeLo^-NLR roles as plasma membrane Ca^2+^ ion channels? It might indicate another sub-cellular function of these NLR modules or a mechanism for controlling levels of ‘active’ resistosome at the plasma membrane, and thus Ca^2+^ influx into cells. Another unanswered question is whether and how different TNL- and TIR-catalysed nucleotides and cyclic nucleotides cooperate in steering defence pathways. Although it will be a challenge to track the accumulation and bioactivities of different nucleotides, it seems reasonable to imagine that combined TNL and TIR enzyme activities and the nature of their substrates might determine immunity and stress resilience outcomes.

## Data Availability

This article has no additional data.
